# Artificial intelligence-assisted type 2 inflammatory endotyping in CRSwNP: from Sinus CT and digital pathology to biologic decision support

**DOI:** 10.3389/falgy.2026.1923079

**Published:** 2026-09-02

**Authors:** Xinou Jiang, Tianhui Kang, Chuan Chen, Hong Qiao, Wei Lv, Aodeng Surita

**Affiliations:** Department of Otolaryngology, Peking Union Medical College Hospital, Chinese Academy of Medical Sciences and Peking Union Medical College, Beijing, China

**Keywords:** artificial intelligence, biologics, chronic rhinosinusitis with nasal polyps, deep learning, digital pathology, multimodal modeling, radiomics, type 2 inflammation

## Abstract

Chronic rhinosinusitis with nasal polyps (CRSwNP) is a heterogeneous rhinologic disease frequently driven by type 2 inflammation, yet routine clinical phenotyping remains insufficient for endotype-informed management. Conventional markers, including blood eosinophils, serum immunoglobulin E (IgE), fractional exhaled nitric oxide, tissue eosinophilia, nasal polyp score, and sinus computed tomography (CT) scores, provide useful but incomplete information. Artificial intelligence (AI) enables integration of sinus CT, radiomic features, digital pathology, biomarkers, comorbidities, and follow-up treatment outcomes. Emerging studies suggest that CT-based AI supports non-invasive identification of eosinophilic or type 2-high disease, whereas digital pathology links routine histology with inflammatory and molecular endotypes. However, published evidence remains dominated by retrospective single-modality studies or partial multi-source integration, and no model has yet jointly integrated sinus CT, digital whole-slide pathology, and biomarkers in CRSwNP. Additional limitations include heterogeneous endotype labels, insufficient external validation, and uncertain clinical utility for biologic decision-making. This Mini Review synthesizes existing single-modality artificial intelligence pipelines, contextualizes CRSwNP as a representative disease model for AI-driven type 2 inflammatory endotyping, and proposes a clinically oriented, multimodal interpretable scoring framework integrating sinus imaging and digital pathology to support precision type 2 stratification in CRS management.

## 1 Introduction: chronic rhinosinusitis with nasal polyps as a clinically accessible model of type 2 inflammation

Chronic rhinosinusitis with nasal polyps (CRSwNP) is one of the most clinically recognizable and biologically heterogeneous phenotypes of chronic rhinosinusitis. In many Western populations and an increasing proportion of Asian patients, it is characterized by type 2 inflammation with eosinophilic infiltration and activation of interleukin (IL)-4, IL-5, IL-13, immunoglobulin E (IgE) and epithelial alarmin signaling (1, 2). The introduction of biologics targeting interleukin-4 receptor *α* (IL-4R*α*), IgE, IL-5/interleukin-5 receptor (IL-5R), and upstream epithelial signals has shifted management from symptomatic surgery to mechanism-directed therapy, making CRSwNP an optimal model for precision rhinology (3, 4).

CRSwNP is also well suited to artificial intelligence (AI)-assisted endotyping, as routine clinical workflows yield multi-dimensional datasets: nasal endoscopy, sinus computed tomography (CT), surgical histology, measurable biomarkers, airway comorbidities and long-term therapeutic follow-up. Conventional phenotypes and single biomarkers incompletely capture this heterogeneity. Multimodal AI integrating radiomics, deep learning and digital pathology can synthesize multi-source data to generate interpretable inflammatory endotype risk scores.

This Mini Review frames CRSwNP as a rhinologic model of type 2 inflammation and summarizes AI tools spanning sinus CT, digital pathology and multimodal biologic decision-making. A targeted literature search was performed in PubMed and the Web of Science Core Collection from January 1, 2020 to June 2026. Four thematic domains were covered: AI-assisted sinonasal CT/imaging analysis, digital pathology/pathomics, multimodal or multi-omics machine learning, and AI-assisted biologic decision support or treatment-response prediction. Search terms combined rhinosinusitis, chronic rhinosinusitis, CRS, CRSwNP, and nasal polyps with AI-related terms, including artificial intelligence, machine learning, deep learning, convolutional neural network, radiomics, pathomics, digital pathology, whole-slide imaging, multimodal learning, multi-omics, ChatGPT, large language model, dupilumab, mepolizumab, omalizumab, and biologics. Eligible studies were peer-reviewed CRS/CRSwNP articles applying AI-related methods to four main domains mentioned above. Studies without an AI component, unrelated to sinonasal disease, conference abstracts, unpeer-reviewed preprints, purely preclinical mechanistic studies, and generic AI studies without rhinologic relevance were excluded. To reduce omission bias, broad keyword combinations were used consistently across both databases, reference lists of relevant articles and reviews were manually checked, and priority was given to studies with larger cohorts, multicenter data, external validation, model comparison, interpretability, and translational relevance. As this was a narrative Mini Review, no PRISMA flow diagram, formal risk-of-bias assessment, or quantitative synthesis was performed.

## 2 The impact of CRSwNP on quality of life and the rationale for AI-assisted type 2 endotyping in rhinology

AI is clinically relevant to CRSwNP because the disease itself is intrinsically multidimensional. In the biologic era, rhinologists must infer inflammatory mechanisms, recurrence risk, biologic eligibility, likely response, and treatment trajectories from symptoms, endoscopy, sinus CT, biomarkers, histopathology, comorbid airway disease, and prior treatment response. Separate analysis of each dataset cannot reliably identify inflammatory endotypes or deliver personalized therapeutic guidance.

### 2.1 Clinical phenotypes do not reliably define inflammatory endotypes

Although CRSwNP is defined by bilateral nasal polyps and characteristic sinonasal symptoms, phenotype does not reliably identify endotype. Patients with matching clinical manifestations vary widely in tissue eosinophilia, local IgE synthesis, neutrophilic infiltration, tissue remodeling and concurrent lower airway disease. Early biomarker-based clustering studies demonstrated that similar phenotypes encompass distinct inflammatory endotypes, including type 1 (T1), type 2 (T2), type 3 (T3), and mixed immune patterns (5). Single-cell and transcriptomic studies further demonstrate immune, epithelial, and stromal heterogeneity within nasal polyps (6, 7). This heterogeneity also extends to aspirin-exacerbated respiratory disease (AERD) and non-AERD CRSwNP, which exhibit distinct immune endotypes and gene-expression profiles, further supporting molecular stratification beyond conventional clinical phenotyping (8). Clinical phenotypes enable preliminary diagnosis yet cannot support mechanism-driven patient stratification.

### 2.2 Conventional biomarkers provide complementary but incomplete endotype information

Conventional biomarkers provide complementary but fragmented information. Blood eosinophils, serum IgE, allergen-specific IgE, and fractional exhaled nitric oxide reflect systemic type 2 inflammation but do not consistently represent local sinonasal disease. Tissue eosinophilia better predicts local disease prognosis yet requires surgical specimens and varies by sampling and counting standards. Clinical and imaging features, including nasal polyp score, Lund-Mackay score, ethmoid-to-maxillary dominance, prior surgery, corticosteroid exposure, asthma, nonsteroidal anti-inflammatory drug-exacerbated respiratory disease (N-ERD), and allergic rhinitis quantify disease severity but cannot forecast biologic response (4, 9). Critically, no single biomarker fulfills three core clinical needs: non-invasive preoperative screening, standardized postoperative endotyping and personalized biologic efficacy prediction. Therefore, AI-assisted inflammatory endotyping acts as an integrated probabilistic tool to synthesize scattered clinical data without replacing rhinologist clinical judgement.

## 3 CT-based AI for non-invasive inflammatory endotyping

### 3.1 Sinus CT as a routinely available substrate for AI-assisted inflammatory endotyping

Sinus CT is standard preoperative imaging for CRSwNP to quantify overall disease burden (10). Although the Lund-Mackay score remains clinically useful (11), it cannot capture high-dimensional information in sinus CT, including spatial distribution, density, texture, osteitis, and ethmoid-to-maxillary patterns (12, 13). AI can transform these features into quantitative biomarkers (14). Emerging evidence identifies CT radiomic signatures correlated with type 2 inflammation (15), enabling non-invasive preoperative inflammatory endotyping.

### 3.2 Radiomics and deep learning for endotyping

The evolution of CT-based AI studies is outlined in Tables 1A,C. Early CT-based artificial intelligence studies in CRSwNP relied on radiomics, which extracts handcrafted intensity, texture and spatial features from manually segmented CT regions (13, 14). In a multicenter study involving 431 patients from three institutions, Zhu et al. developed a radiomics model using 10 imaging features selected by least absolute shrinkage and selection operator (LASSO) regression, achieving an area under the receiver operating characteristic curve (AUC) of 0.815 for identifying eosinophilic CRSwNP, outperforming the conventional ethmoid-to-maxillary ratio model (AUC 0.655) (15). Nevertheless, manual segmentation and laborious feature engineering restrict radiomic reproducibility and clinical scalability (16).

**Table 1A:** Representative AI applications for type 2 inflammatory endotyping and biologic decision support in CRSwNP: summary of AI-based CT studies in rhinology.

Rhinologic problem	Study dataset	AI architecture	AI task	Learning approach	Data augmentation	Validation	Data collection	Study center involvement	Performance comparison	AUC	Key strengths	Translational limitations	Reference
Distinguishing nasal polyps from inverted papilloma	1,791 patients; sinus CT; 1,106 primary-cohort patients from one hospital plus 685 external-test patients from another hospital	3D ResNet, 3D Xception, HRNet	Binary differential diagnosis of NP vs. IP on CT	Supervised	Yes	Holdout internal and external test sets	Retrospective	Multi-center	Both AI to AI and AI to human	0.981 internal; 0.933 external for 3D Xception	Large multicenter CT cohort; external validation; compared with radiologists; explored biological plausibility by proteomics	Retrospective; manual lesion outlining; class imbalance; external validation still limited to two hospitals	(44)
Differentiating NP from IP	296 pathology-confirmed patients; 152 IP and 144 NP; non-contrast CT from two medical centers	Radiomics with SVM, Naive Bayes, XGBoost; feature selection by Boruta, random forest, correlation coefficient	Binary classification of NP vs. IP	Supervised	No	10-fold cross-validation	Retrospective	Multi-center	AI to AI	Best AUC 0.922 with XGBoost	Uses multiple classifiers and feature-selection strategies; pathology-confirmed lesions	Handcrafted radiomics and manual annotation; limited sample size; retrospective; prospective generalizability untested	(45)
Preoperative CRSwNP endotype prediction	192 CRSwNP patients; axial/coronal/sagittal sinus CT; eCRS vs. non-eCRS; single hospital	Lightweight multi-view fusion network/ResMini based on modified residual network	Classification of eosinophilic vs. non-eosinophilic CRSwNP	Supervised	Yes	Holdout test set plus 5-fold cross-validation for model comparison	Retrospective	Single-center	AI to AI	0.991 test set	Uses multi-view CT rather than a single plane; lightweight model; strong discrimination	Single-center; no prospective external validation; surgical CRSwNP population only	(19)
Preoperative CRSwNP endotype prediction	192 CRSwNP patients; 22,265 reported CT images/multi-plane CT groups; Second Affiliated Hospital of Shantou University Medical College	Multichannel ResNet18 with feature adaptive fusion	Classification of eCRSwNP vs. non-eCRSwNP	Supervised	NS	Holdout train/validation/test split	Retrospective	Single-center	AI to AI	0.984 test set	End-to-end multi-plane CT model; high image-level performance; compares single-channel vs. multichannel input	Single-center; no prospective external validation; only patients undergoing surgery	(18)
CRSwNP endotype prediction	251 CRSwNP patients; 29,993 axial/coronal/sagittal sinus CT images; single tertiary hospital	ResNet-18	Classification of eosinophilic vs. non-eosinophilic CRSwNP	Supervised	NS	Holdout train/validation/test split	Retrospective	Single-center	NS	0.963 test set	Uses three CT planes and a larger image set than earlier single-plane studies	Single-center; no external/prospective validation; limited to CRSwNP and surgical cases	(17)
Preoperative differentiation of eosinophilic vs. non-eosinophilic CRS	878 surgical CRS patients; 343 ECRS and 535 NECRS; 56,892 axial sinus CT images; 1,365 images manually annotated for segmentation; ECRS defined as tissue eosinophil/inflammatory cell ratio >=10%	Deeplabv3 for sinus-region segmentation; transferred EfficientNet-B0, ResNet50, Inception-ResNet-v2, and Xception networks for classification	Automatic sinus-region segmentation and classification of ECRS vs. NECRS on preoperative CT	Supervised semantic segmentation and supervised transfer-learning classification	NS	4:1 train/test split; internal test cohort only; models evaluated using both image-level and patient-level labeling	Retrospective	Single-center	AI to AI; patient-level vs. image-level labeling; AI to peripheral blood eosinophil percentage thresholds	Patient-level models: average AUC 0.893, best AUC 0.90; image-level models: average AUC 0.845; Deeplabv3 Dice 0.961	Large CRS CT dataset; fully automatic segmentation before classification; compared multiple CNN architectures; showed patient-level labeling outperformed image-level labeling; DL models outperformed peripheral eosinophil percentage	Single-center retrospective surgical cohort; no external validation; limited to patients undergoing surgery; ECRS definition based on postoperative histology, limiting direct preoperative reference availability	(46)
Objective quantification of sinus opacification in CRS	690 sinus CT scans from a tertiary respiratory hospital; 180 algorithm-development scans and 510 non-overlapping test scans	CNN based on Tiramisu architecture	Sinus segmentation and volumetric opacification quantification	Supervised	NS	Holdout technical validation plus independent test cohort	Retrospective	Single-center	AI to human	N/A; Dice 0.93; Spearman rho 0.82 vs. Lund-Mackay	Fully automated volumetric CT scoring; large test cohort; correlates with visual CT scores and clinical variables	Single-center respiratory-care population; limited manual training set; not prospectively tested	(47)
Quantitative sinus CT assessment in CRS	88 adult CRS surgical subjects; thin-cut CT plus clinical/pathology data from University of Colorado cohort	CNN-based automated sinus segmentation and quantitative CT analysis	Segmentation; quantification of sinus opacification, opacity HU, and osteitis	Supervised	NS	Clinical holdout validation	Retrospective analysis of a prospective cohort	Single-center	AI to human	N/A; rho 0.85 vs. Lund-Mackay; rho 0.48 vs. osteitis score	Extends CT quantification beyond opacification to osteitis and opacity density; clinically characterized CRS cohort	Small single-center surgical cohort; retrospective analysis; needs prospective multi-institutional utility testing	(12)
Automated CRS severity scoring and QoL correlation	445 adult patients from two tertiary centers; sinus CT; 300 train/validation, 74 external test, 71 independent CRS surgical test cases	nnU-Net 3D segmentation model with human-involved model-iteration strategy	Sinus segmentation and derivation of quantitative CT scores	Supervised	NS	Holdout validation, external test, independent test	Retrospective	Multi-center	AI to human	N/A; Dice 0.958 external, 0.871 independent test	External test set; volumetric 3D CT scoring; links automated CT scores with SNOT-22 subdomains	Independent test set was small, same-center, and severe surgical CRS; clinical correlations were modest	(48)
AI CT quantification and olfactory assessment in CRS	84 adult CRS patients prospectively enrolled from three academic centers; sinus CT plus olfactory testing	CNN-based volumetric CT algorithm; Vision Transformer for OMC obstruction	Sinus opacification quantification and OMC obstruction classification	Supervised	NS	Prospective multicenter validation	Prospective	Multi-center	AI to human	OMC AUC 0.79 left, 0.77 right	Multi-institutional prospective validation; connects AI CT outputs with olfactory outcomes	Small cohort; OMC performance moderate; olfaction is multifactorial, limiting CT-only clinical prediction	(49)

Eligible publications retrieved from the targeted literature search were grouped into three categories: AI-based sinonasal CT analyses **(**Table 1A), AI-based digital histopathological studies (Table 1B), and studies using multi-source clinical or paired-modality data (Table 1C). The term “multi-source or paired-modality” does not imply full cross-modal integration. None of the studies summarized in Table 1C jointly integrated sinus CT, digital whole-slide pathology, and biomarkers within a single model. Collectively, the included studies addressed inflammatory endotyping, tissue eosinophilia quantification, type 2-high inflammation, postoperative recurrence risk, and the prediction or assessment of biologic eligibility and treatment response in CRS and CRSwNP. AI, artificial intelligence; AUC, area under the receiver operating characteristic curve; CNN, convolutional neural network; CRS, chronic rhinosinusitis; CRSwNP, chronic rhinosinusitis with nasal polyps; CT, computed tomography; DL, deep learning; eCRS, eosinophilic chronic rhinosinusitis; H&E, hematoxylin and eosin; LLM, large language model; ML, machine learning; N/A, not applicable; NS, not specified; PROM, patient-reported outcome measure; ROC, receiver operating characteristic; SNOT-22, Sino-Nasal Outcome Test-22; WSI, whole-slide image.

These limitations have driven increasing interest in deep learning approaches that automatically learn hierarchical image representations directly from CT data. In one of the earliest endotyping studies, Du et al. developed a residual network-18 (ResNet-18) model using 29,993 CT slices from 251 CRSwNP patients (86 eosinophilic and 165 non-eosinophilic CRSwNP) and achieved a testing AUC of 0.963 for eosinophilic endotype prediction, demonstrating that CT images may encode information reflective of underlying inflammatory biology (17). Building upon this concept, Lai et al. introduced a multichannel ResNet framework incorporating complementary imaging information from different CT planes, achieving an AUC of 0.984 in a cohort of 192 patients (18). Subsequently, Zou et al. proposed a lightweight multi-view fusion network (ResMini) that maintained excellent predictive performance (AUC 0.991) while substantially reducing computational complexity, thereby improving the feasibility of real-world deployment (19). More recently, Li et al. developed and externally validated a multicenter deep-learning model using 1,098 patients from two institutions. By integrating deep learning-derived CT features with clinical characteristics, the model achieved external AUCs exceeding 0.82 and demonstrated improved generalizability compared with previous single-center studies (20). Compared with radiomics, deep-learning models automatically learn hierarchical CT representations and reduce dependence on predefined features. Multi-view or lightweight architectures may improve anatomic coverage and deployment feasibility, whereas CT-clinical models enhance clinical realism by integrating imaging signatures with blood or clinical variables. However, AUCs should be compared cautiously across studies because eCRS/eCRSwNP definitions, eosinophil thresholds, CT inputs, segmentation strategies, and validation designs vary substantially; externally or multicenter-validated models therefore provide stronger translational evidence than single-center models with very high internal performance.

### 3.3 Clinical translation and current limitations

For rhinologists, the greatest value of computed tomography-based artificial intelligence (CT-AI) lies not in replacing conventional radiologic scoring systems but in enabling non-invasive inflammatory endotyping before histopathologic information becomes available. It detects CT signatures linked to tissue eosinophilia and type 2 inflammation, bridging anatomical scans and endotype-directed evaluation to stratify recurrence risk and optimize therapy (17, 18, 20).

Despite promising results, current CT-based AI studies for CRSwNP endotyping have several important limitations. Most published studies are retrospective and single-center, limiting external validity and increasing the risk of selection bias (21). In addition, definitions of eosinophilic CRS and type 2-high disease vary substantially across studies, with inconsistent thresholds for tissue eosinophilia and differing biomarker-based criteria (16). Asian populations carry higher rates of non-type 2 and mixed inflammatory subtypes, limiting model transfer across geographic regions (22–24). Technical heterogeneity in CT scanners, slice thickness, reconstruction algorithms, and acquisition protocols also affects model robustness and reproducibility (25). Finally, although many models report excellent discriminative performance, prospective clinical utility assessments remain lacking. A high AUC for predicting an endotype label should not be interpreted as evidence that a model can directly guide biologic selection, as studies demonstrating improvements in treatment decision-making and patient outcomes are still needed (16).

## 4 Digital pathology and pathomics for spatial type 2 inflammatory signatures

### 4.1 The continuing role and limitations of conventional histopathology

CRSwNP offers a unique advantage: sinonasal tissue is routinely obtainable via surgery or biopsy, allowing histopathological assessment of eosinophilic infiltration, neutrophilic inflammation, basement membrane thickening, edema, goblet cell hyperplasia, glandular changes and fibrin deposition (1). However, conventional pathology faces important limitations: subjective field selection, inconsistent cut-offs for eosinophil counts, spatial heterogeneity within the same polyp, unstable linkage to molecular endotypes, and the fact that routine hematoxylin and eosin (H&E) sections may contain spatial patterns that escape human recognition. These challenges create an unmet need for digital solutions that can standardize and quantify histopathological features in a spatially resolved manner.

### 4.2 Whole-slide imaging, pathomics, and molecularly informed endotyping

The evolution of AI-assisted digital pathology in CRSwNP is summarized in Tables 1B,C. Digital pathology combined with whole-slide image (WSI) analysis is transforming the evaluation of nasal polyp tissue from descriptive histology into quantitative, AI-driven endotyping. Early applications primarily focused on automating tasks traditionally performed by pathologists. The AI chronic rhinosinusitis evaluation platform (AICEP) initially enabled rapid and objective quantification of tissue eosinophilia from H&E slides, improving the efficiency and reproducibility of eosinophilic CRSwNP identification (26). Subsequently, AICEP 2.0 expanded this approach beyond eosinophil counting by simultaneously recognizing eosinophils, neutrophils, lymphocytes, and plasma cells on WSIs, defining four distinct inflammatory subtypes associated with different recurrence risks and outperforming manual high-power field assessment (27). More recently, the nasal polyps subtype diagnosis system (NPSS) framework further extended WSI-based analysis by integrating multidimensional cellular endotyping with three-dimensional reconstruction, enabling visualization of inflammatory cell spatial distributions and improving prognostic prediction for postoperative recurrence (28).

**Table 1B:** Representative AI applications for type 2 inflammatory endotyping and biologic decision support in CRSwNP: summary of AI-based histopathological studies in rhinology.

Rhinologic problem	Study dataset	AI architecture	AI task	Learning approach	Data augmentation	Validation	Data collection	Study center involvement	Performance comparison	AUC	Key strengths	Translational limitations	Reference
Histologic diagnosis of eosinophilic vs. non-eosinophilic CRSwNP	195 nasal polyp WSIs from 3 affiliated hospitals; 179 WSIs for training/internal validation and 16 WSIs for external testing; 26,589 patches	ResNet50, Xception, Inception V3; Inception V3 selected	Prediction of eosinophil ratio on WSI and diagnosis of eCRSwNP	Supervised transfer learning	NS	Internal validation and independent external test	Retrospective	Multi-center	Both AI to AI and AI to human/traditional HPF method	Inception V3: 0.974 internal; 0.957 external	Reduced sampling error from random HPFs; external testing; Grad-CAM supported eosinophil-focused learning	Small external test set; staining/scanning differences may affect generalizability; needs larger multicenter optimization	(26)
Cellular endotyping of nasal polyps	453 patients total; 179 WSIs and 24,625 patches for AICEP 2.0 development; 158-patient prospective cohort and 116-patient retrospective CRSwNP cohort for application	EfficientNet-B5; AICEP 2.0 platform	Quantification of eosinophils, lymphocytes, neutrophils, and plasma cells on WSI; inflammatory phenotype classification	Supervised regression	NS	Train/validation/independent test split; prospective and retrospective application cohorts	Mixed: retrospective model development; prospective and retrospective application cohorts	Mainly single-center	AI to AI	N/A; MAE for eosinophils, lymphocytes, neutrophils, and plasma cells approximately 1.64%, 2.13%, 1.06%, and 1.22%	Quantifies multiple inflammatory cell types and provides heatmaps; identifies clinically meaningful phenotypes	Large WSI file size limits transfer/deployment; generalizability affected by staining/scanning variation; needs broader geographic and non-Asian validation	(27)
Pathological subtype diagnosis and recurrence prediction in nasal polyps	2,457 slides from 20 hospitals for NPSS development; NPSS-MI used 1,047 slides/15,705 MIs; NPSS-WSI used 1,410 slides/21,150 WSI patches; 200-slide clinical comparison with 12 pathologists; 131-patient prospective follow-up cohort	PA-P2PNet for cell detection; U-KAN for region segmentation; NPSS-MI/NPSS-WSI; 3DNP reconstruction	Inflammatory cell detection, region segmentation, NP subtype diagnosis, 3D spatial analysis, and recurrence prediction	Supervised learning; logistic regression with nested CV for recurrence prediction	NS	Internal validation, external test, clinical test, prospective follow-up cohort	Mainly retrospective development; prospective recurrence cohort	Multi-center	Both AI to AI and AI to human	Recurrence prediction: NPSS-WSI AUC about 0.810; WSI + clinical model AUC about 0.826	Large nationwide multicenter dataset; integrates MI, WSI, and 3D reconstruction; improves junior pathologist accuracy and efficiency	One-year recurrence is a practical proxy outcome; needs longer real-world validation; future adaptation to IHC/multiplex pathology needed	(28)
Automated eosinophil quantification in CRS histopathology	Training from 10 WSIs, segmented into 13,324 candidate images, 87,376 negative patches, and 24,374 positive patches; validation in 40 patients/51 WSIs	Query-driven multiple instance learning with hierarchical candidate image selection	Eosinophil detection and eosinophil count estimation on WSI	Weakly supervised MIL	NS	Independent patient validation set	Retrospective	Single-center	NS	N/A; patch-level mean accuracy 87.46%; clinical validation sensitivity 100.00%, specificity 90.48%	MIL addresses heterogeneous eosinophil distribution and reduces HPF selection bias; Grad-CAM visualization	Small number of training WSIs; single-center retrospective evaluation; lacks external validation	(50)
Microvessel quantification and association with type 2 inflammation in CRS	79 CRS patients and 17 controls; 552 H&E images from 27 CRS tissue samples for FCN development; 450 training, 36 validation, 60 test images	Fully convolutional network based on VGG-19	Microvessel segmentation and quantification on H&E images; calculation of MVD and microvessel area ratio	Supervised learning	Yes	Holdout train/validation/test split	Retrospective	Single-center	NS	N/A; test PA 98.81%, MPA 89.01%, mIoU 75.05%	First FCN-based microvessel quantification study in CRS H&E slides; links microvessel area ratio to type 2 inflammation and cytokines	Small single-center dataset; 1–2 slides may not represent whole tissue; manual microvessel labeling still required for training	(51)
Prediction of CRSwNP inflammatory endotypes, gene signatures, and spatial heterogeneity from H&E histology	Prospective multicenter cohort; 100 CRSwNP patients for discovery/internal validation, including 70 training and 30 internal validation cases; 224 patients from 4 hospitals for external validation	EfficientNet-B5 CNN; HE2Signature; patch-level prediction aggregated to WSI-level expression	Prediction of 33 inflammatory signature genes from H&E WSI; T1/T2/T3 endotype classification; spatial heatmap generation	Weakly supervised transfer learning/regression	NS	5-fold cross-validation, internal validation, external validation	Prospective	Multi-center	NS	Internal: T1 0.833, T2 0.903, T3 0.935; external T2 FCER2 + CST1 model AUC 0.716	Predicts molecular endotypes from routine H&E, reducing dependence on transcriptomics; interpretable spatial heatmaps; IHC validation	External performance lower than internal performance; endotype definitions differed between cohorts; requires larger geographically diverse validation	(29)
Prediction of treatment prognosis in CRSwNP from nasal polyp histology	354 CRSwNP patients with H&E-stained nasal polyp WSIs; internal cohort: 232 WSIs split into 185 training/tuning and 47 internal validation WSIs; external cohort: 122 WSIs from 2 hospital	ControlNet ensemble based on three EfficientNet-B5 models with different learning rates; ResNet50 and Inception V3 compared during base-model selection; Grad-CAM for visualization	Prediction of postoperative disease-control status and generation of a poor prognosis score from routine H&E WSIs	Supervised deep learning using treatment outcome labels	NS	Internal holdout validation and independent external validation cohort	Retrospective	Multi-center external validation	AI to AI architecture comparison; AI to conventional tissue eosinophil biomarker	External validation AUC 0.943 for uncontrolled vs. controlled status; AUC 0.891 for uncontrolled vs. controlled/partly controlled; internal validation AUC 0.946 for uncontrolled vs. controlled	Uses routine H&E slides alone; externally validated across two additional hospitals; provides continuous poor prognosis score and interpretable risk heatmaps; outperformed tissue eosinophil percentage; identifies prognostic histologic features including eosinophil infiltration, goblet cell hyperplasia, glandular hyperplasia, squamous metaplasia, and fibrin deposition	Retrospective Chinese cohort; no prospective clinical validation; postoperative control status assessed at non-identical time points; outcome definition partly subjective; scanner/staining variability and cross-regional generalizability remain uncertain; residual black-box limitations	(52)

Eligible publications retrieved from the targeted literature search were grouped into three categories: AI-based sinonasal CT analyses **(**Table 1A), AI-based digital histopathological studies (Table 1B), and studies using multi-source clinical or paired-modality data (Table 1C). The term “multi-source or paired-modality” does not imply full cross-modal integration. None of the studies summarized in Table 1C jointly integrated sinus CT, digital whole-slide pathology, and biomarkers within a single model. Collectively, the included studies addressed inflammatory endotyping, tissue eosinophilia quantification, type 2-high inflammation, postoperative recurrence risk, and the prediction or assessment of biologic eligibility and treatment response in CRS and CRSwNP. AI, artificial intelligence; AUC, area under the receiver operating characteristic curve; CNN, convolutional neural network; CRS, chronic rhinosinusitis; CRSwNP, chronic rhinosinusitis with nasal polyps; CT, computed tomography; DL, deep learning; eCRS, eosinophilic chronic rhinosinusitis; H&E, hematoxylin and eosin; LLM, large language model; ML, machine learning; N/A, not applicable; NS, not specified; PROM, patient-reported outcome measure; ROC, receiver operating characteristic; SNOT-22, Sino-Nasal Outcome Test-22; WSI, whole-slide image.

Recent advances suggest that digital pathology can extend beyond cell enumeration toward molecular endotyping. The HE2Signature model linked H&E WSIs with transcriptomic endotype signatures using interpretable deep learning. Trained on 70 nasal polyp WSIs and externally validated in 224 patients from four centers, it identified T1, T2, and T3 endotypes with AUCs of 0.833, 0.903, and 0.935, respectively, and predicted the T2-associated FCER2/CST1 signature (29). These findings indicate that routine H&E slides contain latent molecular and spatial information that AI can convert into clinically relevant biomarkers for precision rhinology.

Methodologically, these studies show that AI pathology in CRSwNP is moving from automated cell counting toward spatial, prognostic, and molecular inference. Early WSI models mainly reduced sampling bias in eosinophil quantification, whereas later systems expanded to multi-cellular phenotyping, inflammatory subtype classification, and spatial visualization of the tissue microenvironment. More recent models further suggest that routine H&E slides may contain latent prognostic or molecular information that can be extracted by interpretable deep learning.

### 4.3 Clinical translation and current limitations

From a rhinologist's perspective, the greatest clinical value of digital pathology lies in postoperative inflammatory endotyping, as tissue specimens provide direct information on local inflammatory endotypes and tissue remodeling. AI-powered digital pathology extends conventional histopathological assessment by enabling objective quantification of tissue eosinophilia and inflammatory cell composition (27), identifying spatial patterns within the inflammatory microenvironment (28), and potentially linking routine H&E morphology with underlying molecular endotypes (29). These advances position digital pathology as a promising tool for refining postoperative endotyping and recurrence risk stratification in CRSwNP.

Nevertheless, several barriers remain before these approaches can be integrated into routine clinical practice. Standardization of tissue sampling sites, section thickness, H&E staining protocols, scanner specifications, and image quality remains unresolved, while inter-institutional variability in pathology workflows may affect model generalizability (30). Moreover, most AI-based analyses still require pathologist oversight for histological interpretation, validation, and clinical contextualization. Consequently, digital pathology should currently be regarded as a complement rather than a replacement for conventional pathological assessment.

## 5 Multimodal clinical machine learning

### 5.1 Why multimodal AI is more clinically realistic than single-modality AI

Current AI models in CRSwNP are predominantly developed within a single data modality, most commonly CT imaging, digital pathology, or blood biomarkers. While these approaches have advanced sinonasal endotyping, clinical decision-making in CRSwNP inherently relies on integrating information from multiple domains rather than interpreting each modality in isolation. In routine practice, rhinologists simultaneously consider symptoms, endoscopic findings, imaging characteristics, biomarkers, comorbidities, and treatment history when assessing inflammatory burden, estimating recurrence risk, and evaluating suitability for biologic therapy (4, 10).

Therefore, multimodal AI is not only a strategy to improve performance but a framework that mirrors rhinologists' clinical reasoning. By integrating complementary data streams, it may bridge isolated biomarkers and real-world treatment stratification.

### 5.2 Toward an interpretable multimodal research framework

**Figure 1:**
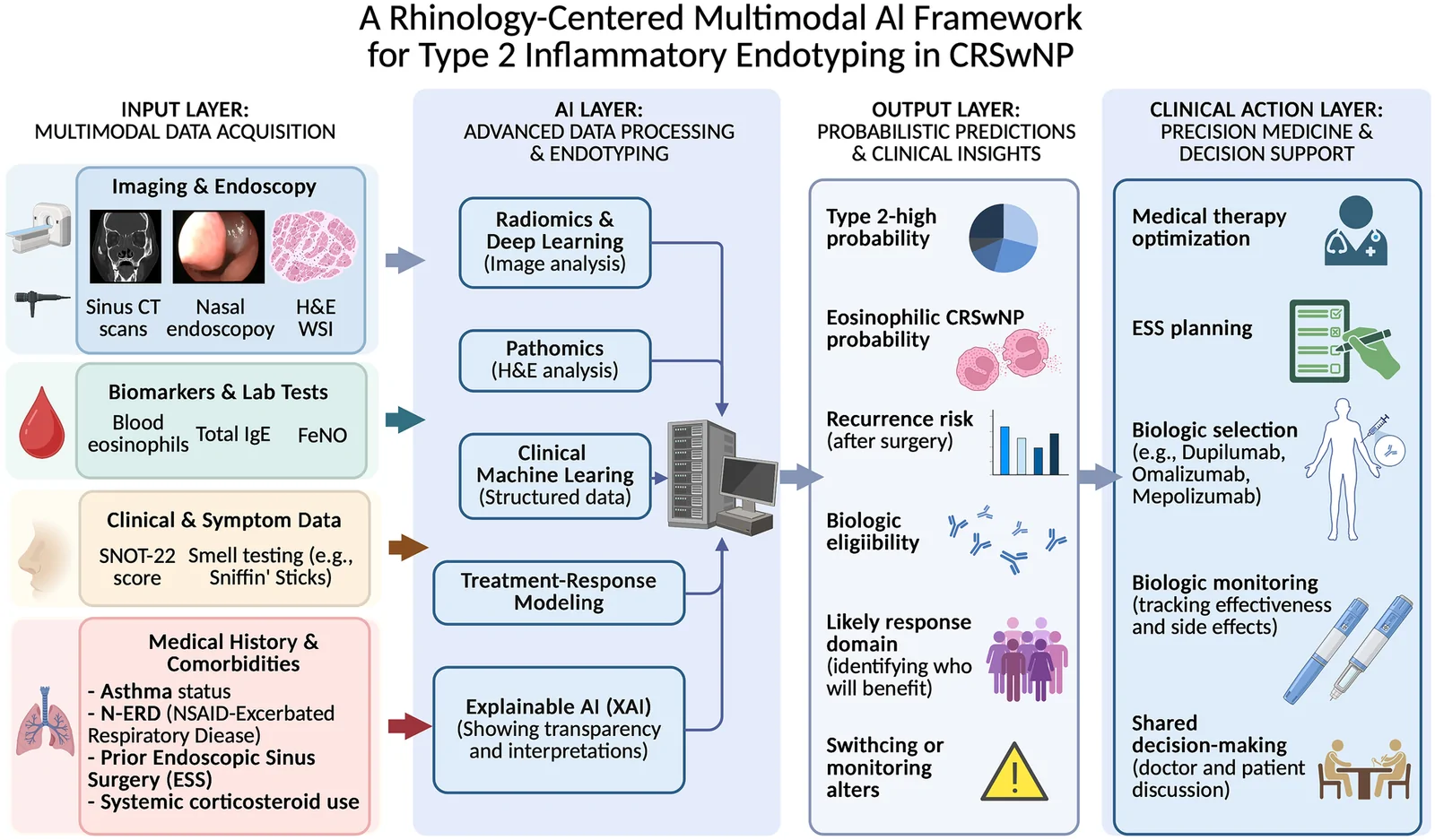
Proposed future multimodal AI research architecture for type 2 inflammatory endotyping in CRSwNP. Routinely available clinical data, sinus CT, nasal endoscopy, H&E whole-slide images, systemic and local biomarkers, comorbidities, treatment history, and longitudinal treatment-response data could be integrated through radiomics, deep learning, pathomics, clinical machine learning, multimodal fusion, and explainable AI. Potential task-specific outputs may include the probability of type 2-high or eosinophilic inflammation, postoperative recurrence risk, biologic eligibility, likely response domains, and monitoring or switching alerts. This figure represents a forward-looking research architecture rather than an operationally defined or validated scoring system. Any resulting outputs should be interpreted as decision-support information for rhinologists rather than autonomous treatment recommendations. AI, artificial intelligence; CRSwNP, chronic rhinosinusitis with nasal polyps; CT, computed tomography; ESS, endoscopic sinus surgery; FeNO, fractional exhaled nitric oxide; H&E, hematoxylin and eosin; IgE, immunoglobulin E; N-ERD, NSAID-exacerbated respiratory disease; NSAID, nonsteroidal anti-inflammatory drug; SNOT-22, Sino-Nasal Outcome Test-22; WSI, whole-slide imaging; XAI, explainable artificial intelligence. (Created in BioRender. Jiang, S. (2026) https://BioRender.com/t97w11z).

Published CRSwNP AI studies have begun to combine selected data sources, but the current evidence represents partial pairwise or multi-source integration rather than full cross-modal modeling (Table 1C). Examples include CT combined with clinical or blood variables (15, 31), imaging phenotypes explored alongside proteomic data (20), and H&E morphology linked to transcriptomic labels (29). These studies demonstrate the potential value of complementary information but do not establish an integrated CT–pathology model. To our knowledge, no published model has jointly integrated sinus CT, digital whole-slide pathology, and biomarkers for CRSwNP inflammatory endotyping. Figure 1 is therefore presented as a proposed future research architecture.

**Table 1C:** Representative AI applications for type 2 inflammatory endotyping and biologic decision support in CRSwNP: summary of studies using multi-source clinical or paired-modality data.

Rhinologic problem	Study dataset	AI architecture	AI task	Learning approach	Data augmentation	Validation	Data collection	Study center involvement	Performance comparison	AUC	Key strengths	Translational limitations	Reference
Preoperative diagnosis of eosinophilic CRS	1,098 patients from two hospitals; sinus CT images plus clinical/blood indicators; 701 training, 79 internal test, 318 external test patients	Integrated model combining 3D-ResNet, 3D-Xception, HRNet outputs with clinical factors via logistic regression/SVM-based integration	Classification of eCRS vs. non-eCRS	Supervised learning	Yes	Fivefold cross-validation in training set; internal and external test sets	Retrospective	Multi-center	Both AI to AI and AI to traditional clinical methods	Integrated model: 0.851 internal; 0.821 external	Large multicenter cohort; independent external validation; integrates complementary CT DL features and clinical information; compared with single DL models and clinical risk factors	Retrospective; surgical population; data from Chinese hospitals only; missing clinical data and staining/diagnostic variability may affect generalizability	(20)
Screening/probable diagnosis of CRS and prediction of CT-confirmed sinonasal inflammation	543 tertiary rhinology-clinic patients; patient-generated health data including demographics, symptoms, prior treatments, comorbidities, PROMs, and CT-derived LMS endpoint	Random forest classifier, deep neural network, XGBoost; logistic regression comparator	Prediction of LMS >= 5 and prediction of probable CRS defined by LMS >= 5 plus >=2 cardinal symptoms	Supervised learning	Yes, SMOTE for secondary endpoint class imbalance	90:10 train/test split with fivefold cross-validation in training	Retrospective	Single-center	Both AI to AI and AI to traditional algorithmic methods	XGBoost: 0.713 for LMS >= 5; 0.798 for LMS >= 5 plus >=2 cardinal symptoms; DNN 0.804 for secondary endpoint	Uses low-cost pre-treatment patient-reported data; may help triage CT use and referral; compares several ML approaches	Single tertiary referral center; modest sensitivity; possible referral and postsurgical bias; needs broader external validation	(53)
Classification of CRS by presence/severity of blood eosinophilia and discovery of key clinical features	399 CRS patients; 65 features including symptoms, SNOT-22/VAS, comorbidities, blood tests, tissue eosinophils, nasal polyp scores, and CT scores	Random forest, XGBoost, logistic regression, SVM; SHAP explainability	Classification of blood eosinophilia presence and severity; identification of key features for CRS eosinophilia classes	Supervised learning	NS	Nested 10-fold cross-validation	Retrospective	Single-center	AI to AI	Setting-1 RF: 0.892 ± 0.037; Setting-2 XGBoost: 0.885 ± 0.022	Uses interpretable ML to reveal basophil count and CT/polyp-score patterns; SHAP provides clinically readable feature importance	Single-center; AEC based on a single blood test; clinical utility of blood-eosinophilia classes remains uncertain; no external validation	(54)
Prediction of eosinophilic CRS	80 CRS surgical patients; peripheral eosinophil count, urinary leukotriene E4, and nasal polyp status; tissue eosinophilia used as reference	Logistic regression and artificial neural network	Classification of eCRS, defined as tissue eosinophils >10/HPF	Supervised learning	NS	Random and surgeon-specific train/test splits; additional ANN cross-validation with one-third holdback	Retrospective	Single-center	AI to traditional biomarkers	Logistic regression: 0.882 random, 0.945 surgeon-specific; ANN: 0.918 random, 0.956 surgeon-specific	Uses easily available clinical/biomarker inputs; ML outperformed individual biomarkers; clinically relevant non-invasive eCRS prediction	Pilot study with small sample size; uLTE4 availability may introduce selection bias; single-center; requires prospective external validation	(55)
Preoperative prediction of eosinophilic CRSwNP using clinical, biomarker, and CT-derived variables	109 adult CRSwNP surgical patients; 60 eCRSwNP and 49 non-eCRSwNP; 60 healthy controls used for baseline comparison; input candidates included demographics, comorbidities, Lund-Mackay score, E/M ratio, blood eosinophils, total IgE, FeNO, and nNO	Artificial neural network with one hidden layer; logistic regression comparator; feature selection using Boruta algorithm plus univariate/multivariate analysis	Classification of eCRSwNP vs. non-eCRSwNP using non-invasive clinical biomarkers and CT score-derived E/M ratio	Supervised learning	No	Random train/test split: 72 training and 37 test patients	Prospective/clinical cross-sectional cohort	Single-center	ANN vs. logistic regression; selected 4-variable models vs. 15-variable models; AI vs. univariate biomarker models	ANN model 1 AUC 0.976 vs. LR model 1 AUC 0.902; ANN model 2 AUC 0.970 vs. LR model 2 AUC 0.845	Integrates readily available non-invasive variables; identifies nNO, peripheral eosinophil absolute count, total IgE, and E/M ratio as key predictors; ANN outperformed LR and single-variable models; clinically practical input set	Small single-center cohort; test set only 37 patients; no external validation; ANN interpretability limited; cross-sectional design; histological eCRSwNP label still based on surgical tissue	(56)
CRS molecular endotype prediction and biomarker discovery	80 patients: 20 disease controls, 20 CRSsNP, 20 CRSwNP, and 20 N-ERD; nasal secretions and serum targeted proteomics plus clinical scores	Targeted proteomics with PCA, hierarchical clustering, Random Forest, SVM, gradient boosting, and SHAP	Identification of CRS endotype-associated protein signatures and biomarker-based classification	Supervised and unsupervised ML	NS	Fivefold cross-validation	Prospective/clinical cohort, exact collection design NS	Single-center	Both AI to AI and AI to clinical variables	Four-class prediction was limited; binary discrimination reached AUC ± 0.01 for SNOT-22 in DC vs. CRSsNP/DC vs. polyps; TPS AUC 0.98 ± 0.02; GDNF and CLC were strongest protein biomarkers	Integrates nasal and serum proteomics with clinical scores; identifies GDNF and CLC as candidate biomarkers; interpretable feature-importance analysis	Small balanced cohort; overlap between CRSwNP and N-ERD limits multiclass classification; age/sex differences and medication effects may bias protein levels; needs larger validation	(57)
Molecular response endotyping and prediction of dupilumab response in airway type 2 disease with CRSwNP/N-ERD	66 whole-blood RNA-seq samples: 18 severe asthma patients before/after 6 months of dupilumab, including 6 with CRSwNP and 12 with N-ERD, plus 30 healthy controls; qPCR validation and clinical response metrics	Unsupervised ML clustering of transcriptomic profiles; qPCR validation; ROC analysis of candidate biomarkers	Discovery of molecular response endotypes and prediction of CRSwNP super-response/non-response	Unsupervised ML plus biomarker-based ROC analysis	NS	Paired pre/post-treatment analysis; qPCR validation; no external validation	Prospective	Single-center	AI to clinical/biomarker response assessment	DIXDC1 baseline expression predicted CRSwNP non-super-response: AUC 0.979; combined gene set AUC 1.0, exploratory	Directly linked transcriptomic changes with dupilumab response; identifies candidate biomarker for CRSwNP non-super-response	Small subgroup size; single-center; near-perfect AUC likely overfitting; whole-blood transcriptomics may not fully reflect sinonasal tissue; needs independent validation	(58)
Preoperative recurrence prediction after CRS surgery	265 CRS patients requiring surgery; preoperative sinus CT plus clinical/laboratory factors and postoperative recurrence follow-up; 200 training patients from center A and 65 external test patients from center B	Multi-task deep learning network based on 3D U-Net; DLR signature combined with clinical factors in a nomogram	Sinus segmentation and post-treatment recurrence prediction	Supervised learning	NS	Stratified fivefold cross-validation and external independent test cohort	Retrospective	Multi-center	Both AI to AI and AI to traditional algorithmic/clinical methods	DLR model: 0.742 external; clinical-radiomic nomogram: 0.842 external	Combines segmentation and prognosis prediction; external test cohort; nomogram improved over clinical model and traditional radiomics	Small and imbalanced external cohort; retrospective; recurrence definition depends on follow-up; needs larger multicenter prospective validation	(59)

Eligible publications retrieved from the targeted literature search were grouped into three categories: AI-based sinonasal CT analyses **(**Table 1A), AI-based digital histopathological studies (Table 1B), and studies using multi-source clinical or paired-modality data (Table 1C). The term “multi-source or paired-modality” does not imply full cross-modal integration. None of the studies summarized in Table 1C jointly integrated sinus CT, digital whole-slide pathology, and biomarkers within a single model. Collectively, the included studies addressed inflammatory endotyping, tissue eosinophilia quantification, type 2-high inflammation, postoperative recurrence risk, and the prediction or assessment of biologic eligibility and treatment response in CRS and CRSwNP. AI, artificial intelligence; AUC, area under the receiver operating characteristic curve; CNN, convolutional neural network; CRS, chronic rhinosinusitis; CRSwNP, chronic rhinosinusitis with nasal polyps; CT, computed tomography; DL, deep learning; eCRS, eosinophilic chronic rhinosinusitis; H&E, hematoxylin and eosin; LLM, large language model; ML, machine learning; N/A, not applicable; NS, not specified; PROM, patient-reported outcome measure; ROC, receiver operating characteristic; SNOT-22, Sino-Nasal Outcome Test-22; WSI, whole-slide image.

Any future implementation should prespecify modality-specific inputs, including CT radiomic or deep-learning features, histological cell-composition and spatial features, systemic or local biomarkers, and clinically relevant covariates. Feature weights should be learned within the development cohort rather than assigned *a priori* and should be locked before external validation. Reference standards should be task-specific, such as a prespecified consensus tissue-defined inflammatory endotype, standardized postoperative recurrence criteria, or validated biologic-response definitions. Missing inputs should be addressed using transparent imputation strategies, missingness indicators, or modality-aware modeling, accompanied by sensitivity analyses. Evaluation should include discrimination, calibration, incremental clinical utility, fairness, and prospective multicenter transportability.

## 6 AI-assisted biologic decision support and monitoring

### 6.1 The challenge of biologic target selection in CRSwNP

Biologic selection in CRSwNP is becoming increasingly challenging as therapeutic options now extend beyond anti-IL-4Rα to include anti-IgE, anti-IL-5/IL-5R, and emerging anti-thymic stromal lymphopoietin (TSLP) strategies (3, 4). However, patients with apparently similar type 2 disease often differ substantially in symptom burden, comorbidities, inflammatory profiles, recurrence risk, and treatment priorities. Moreover, validated predictive biomarkers capable of matching individual patients to specific biologic targets remain limited, and direct head-to-head comparisons between biologics are lacking. Consequently, biologic selection has evolved into a multidimensional decision-making process that requires integration of heterogeneous clinical information rather than reliance on a single biomarker or guideline criterion.

### 6.2 Predictive modeling for eligibility, response domains, and treatment trajectories

Emerging evidence suggests that AI may support several stages of biologic management in CRSwNP. Machine-learning approaches have demonstrated the ability to identify eosinophilic inflammation and type 2 inflammatory endotypes in CRSwNP (15, 29, 31). Because assessment of type 2 inflammatory burden is central to biologic decision-making, these approaches may facilitate identification of patients who warrant consideration for biologic therapy (4). More recently, predictive models have been applied to biologic treatment datasets to estimate treatment response. In a real-world cohort of 84 CRSwNP patients receiving mepolizumab, machine-learning algorithms, particularly extreme gradient boosting (XGBoost), predicted sustained super-response over 24 months with an receiver operating characteristic area under the curve (ROC-AUC) of 0.766, identifying baseline blood eosinophilia, neutrophilia, and AERD comorbidity as important predictors of treatment outcomes (32). These findings suggest that AI may not only enhance inflammatory endotyping but also support prediction of biologic response trajectories using multidimensional clinical data.

### 6.3 Large language models: guideline synthesis rather than autonomous selection

Large language models (LLMs) represent a distinct but rapidly evolving area of interest in biologic decision support. Recent studies evaluating ChatGPT for biologic selection in CRSwNP have reported moderate agreement with expert rhinology recommendations, although substantial variability was observed across different biologic agents (33–35). These findings suggest that LLMs may be useful for synthesizing guideline recommendations, summarizing evidence, and supporting clinician education. However, current evidence remains insufficient to support autonomous biologic selection, particularly given concerns regarding consistency, transparency, accountability, and the absence of patient-specific predictive capabilities. At present, LLMs should therefore be regarded as clinician-facing decision-support tools rather than substitutes for multidisciplinary clinical judgment.

## 7 Validation, explainability, and clinical governance

Despite encouraging performance in imaging, digital pathology, and biologic-response prediction, several challenges must be addressed before AI can be integrated into routine CRSwNP care. First, the lack of standardized reference labels remains a major barrier. Definitions of eosinophilic CRS, type 2-high disease, biologic eligibility, and treatment response vary substantially across studies, limiting comparability and model transportability (36). Moreover, most published models are based on retrospective single-center cohorts, with relatively few undergoing rigorous external validation (37, 38). Future studies should prioritize prospective multicenter validation, calibration assessment, and demonstration of incremental clinical utility beyond existing clinical assessment tools (39). Particular attention should also be paid to biases arising from geographic variation in inflammatory endotypes, imaging protocols, pathology workflows, and patient selection, all of which may affect model fairness and generalizability (40).

AI systems must also be interpretable and compatible with existing clinical workflows. Explainable approaches, including feature importance analysis, attention maps, and biologically informed models, may improve clinician confidence and facilitate validation against established disease mechanisms (41). However, AI should support rather than replace clinician judgment, particularly for biologic selection (42). Robust data governance addressing privacy, transparency, accountability, and data sharing will be essential for responsible implementation (43). Ultimately, successful AI deployment will depend on clinically actionable information that integrates seamlessly into multidisciplinary care.

## 8 Discussion and conclusion

CRSwNP fulfills two principal features that make it a clinically tractable human model of mucosal type 2 inflammation. On one hand, many patients exhibit core molecular, cellular, and tissue-remodeling features of type 2 inflammatory disease, including epithelial alarmin signaling, activation of type 2 innate lymphoid cells and Th2 lymphocytes, tissue eosinophilia, local mucosal IgE production, and characteristic patterns of mucosal remodeling (1, 2, 5–9). Its frequent coexistence with asthma and N-ERD further enables investigation of type 2 inflammation across the united airway. On the other hand, CRSwNP offers substantial clinical research tractability. Polyp tissue obtained during clinically indicated surgery supports multi-omics and spatial pathological analyses, while standardized CT, endoscopy, and serial blood or nasal biomarkers enable longitudinal monitoring. Approved type 2-targeted biologics also permit prospective assessment of pathway-specific treatment responses. Nevertheless, CRSwNP is not a universal surrogate for all type 2 inflammatory disorders, given geographic variation and the presence of non-type 2 and mixed inflammatory endotypes.

The evidence reviewed here suggests that AI in CRSwNP is evolving from isolated classification tasks toward practical clinical decision support. Instead of pursuing high predictive AUC alone, modern AI generates interpretable assessments of inflammatory heterogeneity to guide endotyping, risk stratification and treatment design (42). This evolution mirrors rhinology's shift from clinical phenotype-based diagnosis to precision medicine centered on treatable immune mechanisms.

First, CT-based AI may support non-invasive preoperative estimation of type 2-high endotypes and reduce reliance on postoperative tissue-based assessment. CRSwNP is well suited for CT-based AI research because sinus CT is commonly acquired in routine rhinology visits, delivering high-dimensional texture and spatial information invisible to conventional Lund-Mackay scoring (9). Second, AI-assisted digital pathology may standardize H&E whole-slide inflammatory cell quantification post-surgery, reducing manual counting bias and revealing invisible spatial inflammatory microenvironment patterns. Third, next-generation multimodal AI requires three critical improvements: standardized multicenter datasets, cross-regional prospective validation, and embedded explainable artificial intelligence (XAI) modules for explainable cross-modal fusion. Existing multimodal models generally rely on partial feature-level integration and lack large prospective validation. CRSwNP therefore provides a clinically accessible research context for AI-assisted inflammatory endotyping. Routine rhinology practice readily yields clinical phenotypes, sinus CT images, surgical polyp specimens, systemic biomarkers and follow-up therapeutic data, a complete set of multimodal resources seldom accessible together for other inflammatory diseases. AI's core advantage lies in converting fragmented multi-source clinical data into intuitive risk and stratification indicators to support rhinologists' clinical judgment.

Future work should develop lightweight, externally validated multimodal pipelines compatible with hospital workflows, integrating CT, digital pathology, lab biomarkers and endoscopic data to achieve unified cross-center inflammatory endotyping.
